# Ovarian Aging: A Multifaceted Perspective on Mechanisms

**DOI:** 10.1111/cpr.70144

**Published:** 2025-11-04

**Authors:** Xiaoqing Zhang, Xinyi Dong, Xue Zhang, Suying Yuan, Qiran Zhang, Zilu Guo, Xiaoxue Yang, Qionghua Wang, Tianlan Yang, Donghui Huang

**Affiliations:** ^1^ Institute of Reproduction Health, Tongji Medical College Huazhong University of Science and Technology Wuhan China; ^2^ Maternal and Child Health Centre of Linxiang District of Lincang City Lincang China; ^3^ Shenzhen Huazhong University of Science and Technology Research Institute Shenzhen China

## Abstract

A mechanistic network of ovarian aging, highlighting mitochondrial dysfunction as a central hub interconnected with genetic, metabolic, and inflammatory pathways.

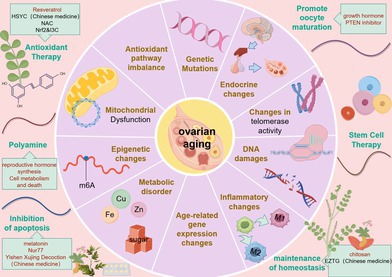

To the editor,

1

Aging is a spontaneous and inevitable process in biological organisms over time. Recent research in the field of cell biology suggests a novel perspective on the role of the vascular system in the aging process [1]. Mu L. found that the aging process of ovarian vascular endothelium precedes that of nonreproductive organ vascular endothelium [2]. This phenomenon contributes to the ovarian tissue's position as the primary human tissue to manifest substantial age‐related functional deterioration [2]. Ovarian aging has been demonstrated to directly initiate the aging process in multiple systems and organs throughout the body [2]. Conversely, research findings suggest that for every 1‐year delay in menopausal age, there is a 2% decrease in all‐cause mortality [3]. Consequently, ovarian aging is regarded as the primary indicator of female bodily aging, influencing the aging of multiple organs.

Ovarian aging is a complex biological process closely linked to human reproductive capacity and population size. It is characterised by the gradual decline of ovarian function with advancing age, accompanied by follicular depletion, diminished egg quality and changes in menstrual cycles, ultimately leading to loss of fertility and menopause [2]. Traditional research has focused on follicular depletion and endocrine changes, while recent evidence reveals complex interactions among mitochondrial dysfunction, epigenetic alterations, metabolic disorders and immune microenvironment remodeling [4, 5, 6, 7, 8]. We propose that mitochondrial dysfunction, by affecting energy metabolism and oocyte quality, represents a core driver of ovarian aging. By reviewing the latest research advances in these mechanisms, elucidating their interactions and identifying potential therapeutic targets, we aim to provide a scientific foundation for addressing ovarian aging.

## 2 Mechanism

### 2.1 Mitochondrial Dysfunction

Extensive research indicates that mitochondrial DNA damage plays a pivotal role in ovarian aging. Mitochondrial DNA is devoid of histone protection, and electron leakage in the mitochondrial respiratory chain renders it vulnerable to oxidative stress damage from reactive oxygen species (ROS) [6]. Chiang L. J. et al. discovered that oxidative mitochondrial damage results in a decrease in mitochondrial DNA copy number and function in the ovary [6]. This, in turn, induces ovarian aging and follicular atresia [6]. The relevant mechanism can be seen in Figure S1. During the process of ovarian aging, a dysregulation of the NAD+/SIRT2 pathway has been observed to cause an imbalance in antioxidant pathways, resulting in a disruption of mitochondrial energy homeostasis [9]. As the ovary undergoes the process of aging, there is an increase in the expression of the NAD‐degrading enzyme CD38 [9]. This increase in expression leads to a decrease in NAD+ levels and a weakening of SIRT2 deacetylase activity [9]. This results in a disruption of mitochondrial energy homeostasis, an inhibition of PINK1/Parkin‐mediated mitochondrial autophagy, and a simultaneous impairment of PARP1‐mediated base excision repair [9]. This metabolic disorder has been shown to trigger an accumulation of mitochondrial reactive oxygen species (mitoROS), which in turn exacerbates egg quality decline through the process of oxidative damage to mitochondrial DNA [9]. Consequently, energy metabolism disruption and mitochondrial oxidative stress resulting from mitochondrial dysfunction represent key mechanisms directly promoting oocyte aging.

### 2.2 Genetic Mechanisms

The DNA damage response is a critical process for maintaining genomic stability. Studies have shown that abnormalities in the function of DNA damage repair (DDR)‐related genes in women during the early stages of natural menopause significantly impact the decline in ovarian reserve [10]. Notably, the substantial age‐related decline in the expression levels of critical repair factors, such as BRCA1 and the meiosis‐specific factor DMC1, within oocytes plays a pivotal role, particularly in accelerating follicular atresia and the depletion of ovarian reserve function [11]. This decline in efficiency is associated with aging, leading to the activation of the p53‐p21 pathway. This, in turn, results in the apoptosis of granulosa cells (GCs) and follicular atresia [11].

### 2.3 Epigenetic and Posttranslational Modifications (PTM)

#### 2.3.1 Lysine Succinylation

Many studies have demonstrated that PTM can influence gene expression or protease activity, thereby modulating cellular functions [7]. Consequently, they have been shown to play a pivotal role in the regulation of ovarian function and the development of associated diseases. Lysine succinylation (Ksuc) is a novel PTM that is widely conserved in eukaryotic and prokaryotic cells and has been implicated in a variety of physiopathological processes, including cell metabolism and tumourigenesis [7]. Mei‐Ling Le et al. found that the higher molecular weight and two negative charges of the succinyl acyl group of Ksuc, compared to conventional PTMs, allow Ksuc to have a greater influence on the properties of proteins [7]. Furthermore, it was found that the Ksuc candidate regulatory enzyme KAT2A showed an age‐dependent increase in the mouse ovary, leading to an imbalance in the level of Ksuc modification in somatic cells, thereby promoting apoptosis of ovarian GCs, impairment of mitochondrial function, reduction in somatic coefficient of ovulation, anti‐Müllerian hormone (AMH) levels, inhibition of GC proliferation and promotion of follicular atresia [7].

#### 2.3.2 Histone Acetylation Modification (HAT1)

Histone Acetyltransferase 1 (HAT1) is a member of the HAT family. It has been demonstrated that HAT1 in GCs is implicated in multiorgan senescence, a process that is critical for mammalian development and genomic stability [12]. Bichun G. et al. have shown that a decrease in HAT1 expression with age is a potential cause of ovarian senescence, and that the mechanism involves defective meiotic conditions in the oocyte and the phenomenon of aneuploidy [12].

Bichun G. et al. found that HAT1 was highly expressed in young rat GCs and significantly decreased in old GCs [12]. Further studies showed that HAT1 acetylates FoxO1, leading to FoxO1 translocation to the nucleus and increased expression of amphoteric trypsin, which plays an important role in oocyte meiosis, thereby reducing oocyte meiotic defects and aneuploidy [12]. Therefore, a gradual decrease in HAT1 expression in somatic cells with age leads to an increase in meiotic defects and aneuploidy in oocytes. Therefore, how to regulate increased HAT1 expression may be a research target to delay ovarian aging.

Other epigenetic factors may influence ovarian aging, and research has also indicated that genetic mutations are associated with ovarian aging. The mechanisms are detailed in Section 3 of the Supporting Information.

### 2.4 Telomerase Activity Regulates the Process of Ovarian Aging

Telomeres are repetitive DNA sequences located at the ends of eukaryotic chromosomes that serve to protect the ends of chromosomes, prevent fusion and disintegration and are essential for maintaining the integrity of genomes and chromosomes [13]. Many studies have shown that the process of ovarian aging is accompanied by a decline in telomere length and structural damage, resulting in a concomitant decrease in telomerase activity and an increase in telomerase reverse transcriptase expression within the follicular GCs [13]. Kosebent et al. found that the expression of telomerase reverse transcriptase catalytic subunit (TERT) and telomerase RNA template (TERC) decreased significantly with age, leading to the inability to lengthen telomeres [13]. Furthermore, they determined that telomere dysfunction was exacerbated by dysregulation of telomere‐associated proteins (e.g., TRF1/2, POT1) [13]. In addition, the study found that follicular cells (especially GCs) experienced a continuation of telomere shortening during cell division [13]. Shortened telomeres are recognised as “broken DNA” and activate the p53‐p21 pathway, which induces cell cycle arrest or apoptosis [13]. GC apoptosis has been demonstrated to result in the destruction of the follicular microenvironment and the loss of oocyte support, ultimately leading to follicular atresia and a reduction in ovarian reserve [13].

### 2.5 Endocrine Changes

Notably, HPO axis dysfunction has been observed to expedite the process of follicle depletion, and the subsequent reduction in ovarian hormone secretion has been shown to negatively regulate HPO axis function, thereby establishing a self‐perpetuating cycle [4]. In addition, this dysregulation of the HPO axis leads to a loss of negative feedback regulation of the ovary by the pituitary gland, which triggers a dramatic increase in follicle‐stimulating hormone (FSH), accelerating the hardening of the follicular microenvironment [4]. The findings, when considered collectively, suggest that HPO axis dysfunction is not merely a consequence of ovarian aging but also a significant contributor to this process.

### 2.6 Metabolic Disorder

#### 2.6.1 Iron Accumulation

A plethora of studies have demonstrated that, with age, iron levels increase in senescent ovaries and oocytes [8]. Furthermore, the expression of iron metabolism proteins is abnormally expressed [8]. Chen Y. et al. found that senescent oocytes exhibited increased ferritin and filament phagocytosis, as well as sustained increases in cell membrane Fe2+, increased lipid peroxidation, mitochondrial dysfunction and increased lysosomal activity, leading to a disruption of ovarian iron homeostasis associated with senescence [8]. Iron overload leads to lipid ROS accumulation, decreased ovarian reserve and oocyte quality through inhibition of glutathione peroxidase 4 (GPX4) and activation of long‐chain lipoyl coenzyme A synthetase 4 (ACSL4) [8]. The relevant mechanism can be seen in Figure S2.

#### 2.6.2 Reduced Ovarian Steroid Synthesis Due to Mitochondrial Dysfunction

Research indicates that the decline in mitochondrial function during the aging process significantly impacts the efficiency of steroid hormone synthesis. LONP1 is a pivotal protein in maintaining mitochondrial protein homeostasis and energy metabolism [14]. Yi Chen et al. demonstrated that the expression levels of the mitochondrial protease LONP1 decrease with age, leading to reduced degradation of its substrate CYP11A1 [14]. CYP11A1 is the key enzyme in the conversion of cholesterol to pregnenolone [14]. Its abnormal accumulation feedback inhibits the initial steps of steroid synthesis, resulting in mitochondrial abnormalities, a reduction in follicle number and oocyte quality, and ultimately, premature depletion of the ovarian reserve [14]. Furthermore, LONP1 interacts with the apoptosis‐inducing factor AIFM1 [14]. In the absence of LONP1, AIFM1 translocates to the nucleus and triggers oocyte apoptosis [14]. Miller et al. also demonstrated that reduced expression of the StAR protein (steroid synthesis acute regulatory protein) on the inner mitochondrial membrane decreases the efficiency of cholesterol transport from the outer to the inner mitochondrial membrane, further limiting the supply of raw materials for steroid hormone synthesis [15]. Therefore, mitochondria have been demonstrated to play a pivotal role in the regulation of steroidogenesis, particularly within the context of the ovary.

Furthermore, the accumulation of advanced glycation end products (AGEs), lipid metabolism disorders, abnormal amino acid metabolism and abnormal nucleotide metabolism has been demonstrated to promote ovarian aging. For specific mechanisms, please refer to Section 4 of the Supporting Information.

### 2.7 Immune and Inflammatory Microenvironment

It has been shown that with age, excessive proliferation of senescent ovarian fibroblasts and accumulation of extracellular matrix lead to a microenvironment that becomes rigid and fibrotic [16], Correspondingly, inflammation‐related pathways are activated and the expression of pro‐inflammatory factors is increased in aging ovaries, creating an inflammatory microenvironment that is likely to induce oocyte damage [5]. Chuanchuan Z et al. discovered that as age progresses, there is an escalating activation of the apoptotic pathway in macrophages (MFs), resulting in a transformation of the ovarian microenvironment into a pro‐inflammatory state [17]. This phenomenon has been demonstrated to promote stromal cell senescence and accelerate reproductive decline [17]. Furthermore, the process of late‐stage ovarian aging is accompanied by an enhancement of the M2 macrophage phenotype, which ultimately leads to the development of ovarian fibrosis [17]. The relevant mechanism diagram can be seen in Figure S3, and the synergistic effect of other immune cells can be found in Section 5 of the Supporting Information.

### 2.8 Mechanism Network Forecasting

Based on these mechanisms, we predicted a network of stage‐specific mechanisms of ovarian aging. We suggest that mitochondrial dysfunction and DNA damage due to the accumulation of ROS in the mitochondria play a dominant role in the early stages of ovarian aging [6], triggering a BRCA1‐dependent DDR mechanism [11]. Mutations in the BRCA1 gene significantly affect the normal physiological processes at this stage [11], resulting in translation postediting anomalies, epigenetic abnormalities and changes in gene expression, including imbalances in the levels of Ksuc modification, down‐regulation of HAT1 expression and so on [7, 12]. In addition, irreversible shortening of telomeres may likewise be an important driver [13].

The onset of perimenopause marks the transition to the mid‐stage of ovarian senescence. The dominant mechanism in this phase may be the dysregulation of the HPO axis and the elevation of FSH and activation of TGF‐β1 signalling due to mutations in FSHR [4]. This, in turn, causes inflammation and the gradual dominance of the M2 macrophage phenotype [17]. An increase in the M2 phenotype can further lead to ovarian fibrosis [17]. ROS‐activated inflammatory vesicles may also be one of the triggers of ovarian fibrosis [8]. Postmenopause is characterised by the late stage of ovarian senescence. The prevailing mechanism during this phase is believed to be metabolic disturbances, encompassing the accumulation of iron and so on [8, 9]. These disturbances, if left unaddressed, may progressively result in the depletion of follicular reserve.

In this mechanistic network, we posit that mitochondrial dysfunction, which affects energy metabolism and oocyte quality, may act as a pivotal catalyst for ovarian aging. This is because the energy crisis and ROS accumulation due to declining mitochondrial function are important triggers of DNA damage, epigenetic disorders and hardening of the inflammatory microenvironment [6]. Furthermore, NAD+ and SIRT family proteins (SIRT2/3) function as pivotal hubs, integrating energy metabolism, epigenetics and inflammation [9]. A decline in NAD+ has been demonstrated to inhibit mitochondrial function via SIRT3, while concomitantly reducing PARP1‐mediated DNA repair [9]. This, in turn, results in a further depletion of the NAD+ pool, thereby establishing a self‐perpetuating cycle [9]. Consequently, a decline in NAD+ and SIRT family proteins results in a synchronised decline in energy metabolism, genomic stability and the immune microenvironment.

## 3 Therapeutic Target for Ovarian Aging

Current strategies for the purpose of delaying ovarian aging include stem cell therapy, antioxidant supplementation and hormone replacement therapy (HRT). Mesenchymal stem cells (MSCs) are known to possess multipotent differentiation capabilities, and they can be isolated from various tissues, including the umbilical cord, placenta and bone marrow [18]. The paracrine signaling and microenvironmental regulation exhibited by MSCs have been demonstrated to possess the potential to restore ovarian function [18]. The fundamental mechanism of HRT in treating ovarian aging involves improving the ovarian microenvironment and delaying follicular depletion through exogenous hormone supplementation [19]. Resveratrol and other antioxidants have also been shown to mitigate oxidative damage and enhance oocyte quality [20]. Measures to delay ovarian aging are detailed in Section 6 of the Supporting Information.

## 4 Conclusion

**FIGURE 1:**
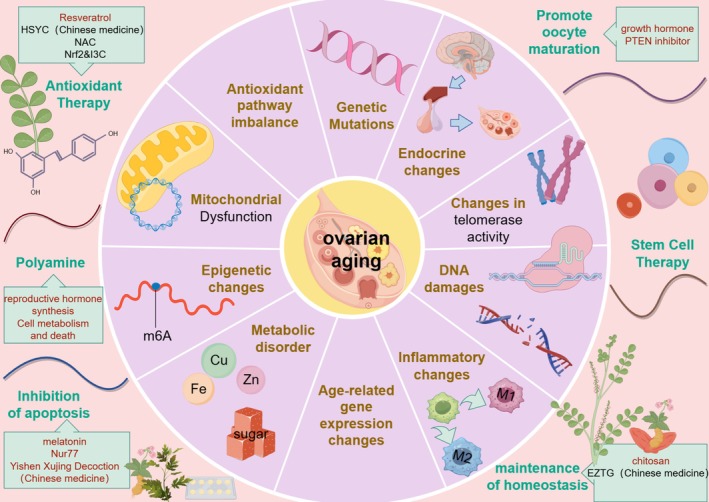
Summary chart. (By Figdraw). The mechanisms of ovarian aging are multifaceted, including, but not limited to, mitochondrial dysfunction, genetic mutations, imbalances in antioxidant pathways, endocrine changes, DNA damage, telomere shortening, epigenetic changes, metabolic disorders and inflammatory changes. These mechanisms interact to form a complex network that leads to ovarian aging.

This letter systematically elucidates the underlying mechanisms and therapeutic approaches of ovarian aging, as illustrated in Figure 1. These mechanisms include mitochondrial dysfunction, diminished DDR capacity and telomerase activity, epigenetic alterations (e.g., m6A modification, imbalance of Ksuc modification), metabolic disorders (iron accumulation, increase in AGEs), alterations in the inflammatory microenvironment (macrophage polarisation), endocrine dysfunctions (dysfunction of the HPO axis) and genetic mutations, among numerous other factors. We hypothesise that mitochondrial dysfunction is a primary trigger, with inflammation, fibrosis, metabolic imbalance and epigenetic alterations likely serving as key drivers of this process. Contradictory findings among several studies are noted, and corresponding rational explanations alongside current research limitations can be found in Section 7 of the Supporting Information.

In summary, ovarian aging is the result of synergistic multimechanisms, but it is important to note that many of the study's key findings are still in the preliminary stages. Future research needs to integrate stage‐specific interventions and precision medicine strategies to prolong the female reproductive lifespan.

## Author Contributions

**Xiaoqing Zhang:** conceptualisation, investigation, writing – original draft, writing – review and editing. **Xinyi Dong:** writing – review and editing, formal analysis. **Xue Zhang:** writing – review and editing, formal analysis. **Xiaoxue Yang:** writing – review and editing, formal analysis. **Suying Yuan:** investigation, methodology. **Qiran Zhang:** investigation, methodology. **Zilu Guo:** investigation, methodology. **Qionghua Wang:** validation, supervision. **Tianlan Yang:** validation, supervision. **Donghui Huang:** validation, supervision. All authors read and approved the submitted version.

## Conflicts of Interest

The authors declare no conflicts of interest.

## Supporting information


**Data S1:** cpr70144‐sup‐0001‐Supinfo.docx.

**Figure S1:** Mechanisms of mitochondrial disorders leading to ovarian senescence. (By Figdraw).

**Figure S2:** Iron metabolism contributes to ovarian aging. (By Figdraw).

**Figure S3:** The process of macrophage polarisation in response to specific danger signals within the aging ovary. (By Figdraw).

## Data Availability

Data sharing not applicable to this article as no datasets were generated or analysed during the current study.
