# Supplementary material for: Ovarian Aging: A Multifaceted Perspective on Mechanisms
Source: Cell Prolif. 2025 Nov 4;59(1):e70144. doi: 10.1111/cpr.70144 (PMC12774617; doi:10.1111/cpr.70144)
Supplement: Supplementary file 1 — Data S1: cpr70144‐sup‐0001‐Supinfo.docx. [file CPR-59-e70144-s001.docx]

**Section 1 Stages of ovarian aging**

The STRAW + 10 segmentation system of the ovarian aging process was presented at the STRAW + 10 Workshop 2011 and consists of three major and ten minor stages^1^. The initial important phase is the growth period, followed by the transition to menopause and the postmenopausal period^1^. It was additionally observed that the perimenopausal period (stages -2 to +1a) serves as a crucial indicator of the physiological changes associated with the aging of the ovary that women are entering^1^.

Unlike sperm, the number of follicular reserves is determined before birth, meaning that germ cells in the ovaries cannot replenish it, and each subsequent ovulation after puberty means fewer follicles^2^. Studies have demonstrated that the initial supply of primordial follicular reserve at birth varies from 10^5^ to 10^6^ follicles^3^, and only one growing follicle survives to ovulate each month throughout the reproductive years up to the menopausal transition (MT)^4,5^. M. Depmann et al. found a positive correlation between the initial supply of primordial follicles in girls at birth and the overall duration of ovarian function in adulthood^6,7^. Sean D. Lawley et al. found that individual primordial follicles are allowed to enter the cell cycle only after a combined stress response checkpoint, such as physiological stress or DNA damage, has been resolved in order to trigger growth activation of primordial follicles^8^. Consequently, in women with a primordial follicle reserve of tens of thousands or more, numerous primordial follicle growth activations are initiated on a daily basis^8^. This phenomenon stands in contrast to scenarios where the reserve undergoes a gradual decline, resulting in the absence of new follicle growth for multiple consecutive days^8^. Consequently, Sean D. Lawley et al. concluded that MT corresponds to a period of seven consecutive days without new follicular growth, followed by a period of twelve consecutive days without new follicular growth^8^, i.e., when the follicular reserve drops from 2000 to approximately 1000. The onset of menopause is characterised by the absence of new follicular activation for a duration exceeding 12 consecutive days^8^, leading to a follicular reserve of mere hundreds.

**Section 2 Mitochondrial Mechanism Diagram Supplement**

The mechanism by which mitochondrial dysfunction influences ovarian aging is illustrated in Supplementary Figure 1. The absence of histones in mitochondrial DNA, in conjunction with the escalating electron leakage from the mitochondrial respiratory chain that occurs with advancing age, renders the mitochondria more vulnerable to oxidative stress triggered by elevated levels of ROS. This, in turn, engenders mitochondrial dysfunction and disrupts energy metabolism. This, in turn, gives rise to two distinct consequences: firstly, follicular atresia and impaired growth; and secondly, damage to the ovarian microenvironment. The net effect of these processes is accelerated follicular atrophy, reduced oocyte quality, and ultimately, ovarian failure.

**Section 3 Genetic Mechanisms Supplement**

**3.1 Epigenetic and Post-Translational Modifications**

**3.1.1 N6-methyladenosine methyltransferase METTL14**

RNA modification is defined as the process of chemically modifying an RNA molecule in such a way that it affects the structure, stability and function of the RNA molecule. A prevalent RNA modification is methylation, which involves the addition of a methyl group to an RNA molecule. m6A, also known as N6-methyladenosine, is a significant epigenetic component that plays a crucial role in various physiological and pathological processes^9^. M6A modification plays a critical role in regulating several aspects of RNA metabolism, including RNA stability, splicing, translation, and degradation. In the ovary, m6A has been implicated in the maturation of oocytes, the regulation of germ cell maturation and development, the synthesis of reproductive hormones and signal transduction^10^.

Jiang Z et al. found that the down-regulation of the m6A demethylase FTO in GCs of senescent ovaries leads to an increase in m6A^9^. This imbalance in m6A modification in somatic cells ultimately leads to GC-mediated ovarian senescence^9^. Notably, m6A modification is reversible^11^. Qian C et al. showed that the dynamic modification of m6A during the maternal-to-homozygous transition is dependent on the regulation of the m6A methyltransferase complex^12^. It was found that METTL14 mRNA levels were significantly elevated in senescent ovaries, with increased expression of the m6A methyltransferase METTL14 and significantly higher levels of m6A modification^12^. METTL14-mediated m6A modifications are preferentially enriched in the 3'UTR of cell cycle-associated mRNAs, promoting YTHDF2-dependent degradation^9^. Li L. et al. demonstrated that in GCs, FTO deficiency leads to m6A overaccumulation and inhibits MMP2 translation^13^. Consequently, it can be deduced that FTO impedes GC-mediated ovarian senescence, and conversely, an augmentation in m6A ultimately results in ovarian senescence.

**3.1.2** **mRNA stability regulation (DCP1A & SPDL1)**

Abnormal expression of the mRNA decapitation enzyme (DCP1A) and the spindle localization protein (SPDL1) has been found to epigenetically regulate ovarian senescence. Kong et al. demonstrated that the promoter or enhancer regions of the DCP1A and SPDL1 genes in senescent oocytes may be aberrantly methylated and modified with histone deacetylation, resulting in up-regulation of DCP1A expression and down-regulation of SPDL1 expression.DCP1A overactivation has been demonstrated to accelerate maternal mRNA degradation, while the down-regulation of SPDL1 expression has been shown to lead to incorrect assembly and aneuploidy.SPDL1 expression was found to be down-regulated in these cases^14^. The over-activation of DCP1A has been shown to accelerate the degradation of maternal mRNA, whereas the down-regulation of SPDL1 expression has been shown to result in spindle assembly errors and an increase in the proportion of aneuploid oocytes^14^. This finding indicates that abnormal epigenetic changes of DCP1A & SPDL1 in oocytes during aging result in a decline in oocyte quality and, consequently, ovarian senescence.

**3.2 Genetic mutation**

**3.2.1 MDR-1**

Multidrug resistance transporter-1 (MDR-1) is an ATP-dependent transmembrane effluxer present in the mitochondrial membrane of oocytes. A substantial body of research has demonstrated that it protects female gametes from oxidative stress and represents a pivotal nexus of metabolite regulation, oxidative stress and mitochondrial dysfunction^15^. Shukla P et al. found that oocyte mutations in the MDR-1 gene encoding the mitochondrial ABC transporter may result in increased susceptibility to Cyclophosphamide-induced ovarian dysfunction and infertility were increased^16^. Dalileh Nabi et al. demonstrated that MDR-1 mutations result in abnormal mitochondrial homeostasis and increased susceptibility to damage from ROS accumulation, leading to reduced oocyte mass and decreased ATP levels^15,17^. These findings collectively underscore the pivotal role of MDR-1 in mitochondrial physiology and elucidate the impact of MDR-1 on oocyte quality and ovarian aging^17^.

**3.2.2 FSH&FSHR**

The main role of FSH is in reproduction, where it induces the production of oestrogen in women and has an important regulatory function^18,19^. Bhartiya D et al. showed the presence of two distinct stem cell populations in the ovarian surface epithelium, including very small embryonic-like stem cells (VSELs) and ovarian stem cells (OSCs), which are responsible for neogenesis and the assembly of the primordial follicle in adulthood and are regulated by FSH through its alternative splice receptor variant FSHR3^20^. Zaidi M, Broekmans FJ, Tiwari-Pandey R et al. found that during the process of ovarian aging, there is a gradual decrease in the antral follicular population and somatic mutations in the FSH receptor (FSHR) expressed on GCs. These changes lead to a dramatic increase in serum FSH^21–23^. Yanye Z et al. found that high serum FSH levels lead to a reduction in ovarian reserve through a mechanism involving the promotion of ovarian fibrosis by the FSHR/TGF-β1/SMADS pathway^24^. Their study showed that FSH treatment increases the phosphorylation levels of SMAD2/3 and SMAD4 and activates the TGF-β1 signaling pathway, leading to nuclear translocation of SMADs complexes in MOFs and increased expression levels of fibrotic molecules in MOFs^24^. This finding indicates that an increase in FSH results in a decrease in follicle numbers and accelerated ovarian fibrosis.

**Section 4 Metabolism-Related Mechanisms Supplement**

**4.1 Iron accumulation**

Ma J et al. found that reduced Cry1 expression in senescent ovarian granulosa cells impairs NCOA4-mediated ferritin phagocytosis, leading to increased cellular ferritin uptake and subsequent granulosa cell senescence^25^. As shown in Supplementary Figure 2, Chen Y et al. observed that senescent oocytes exhibit sustained increases in membrane Fe²⁺, mitochondrial dysfunction, and elevated lysosomal activity, resulting in iron accumulation^26^. Furthermore, Schröder SK et al. found that in Esr1-deficient ovaries, the iron homeostasis regulator lipoprotin 2 was highly expressed, iron metabolism was altered, and iron accumulation increased, leading to a significant increase in ovarian mast cells, which participate in iron-mediated foam cell formation^27^.

**4.2 Disorder of glucose metabolism**

The interaction of biomolecules with ROS generates deleterious products that further induce oxidative damage in the ovarian microenvironment, for example, advanced glycation end products (AGEs)^11^. Nan Zhu found that AGEs have been shown to accumulate in long-lived tissues and organs with age, leading to hyperactivation of the primordial follicle pool, direct cross-linking with proteins to cause deformation and inactivation of related proteins, and binding to receptors to form an AGEs-RAGE axis to mediate oxidative and carbonyl stress in cells, leading to premature depletion of the follicle pool^28^. Shanlini Mani et al. foun-8d that the interaction between AGE and RAGE receptors may also affect oocyte quality^11^. Furthermore, AGE interacts with ROS to activate pro-inflammatory pathways, impair oocyte quality, induce oxidative damage to the ovarian microenvironment, and exacerbate oxidative stress in the ovary^11^. Additionally, AGE may influence primordial follicle activation by affecting a range of cellular responses, including autophagy and inflammatory damage, and triggering the PI3K-PTEN signalling pathway, thereby accelerating the onset of ovarian aging^28^.

**4.3 Disorder of Lipid Metabolism**

There is a demonstrable correlation between imbalances in lipid metabolism and the decline in ovarian function. Wu et al. discovered that aberrant expression of the glycerophospholipid metabolism enzyme GPD1L during the process of ovarian aging results in lipid metabolism disorders within the follicular fluid, consequently impacting follicle development and oocyte quality^29^. Furthermore, iron overload has been shown to inhibit glutathione peroxidase 4 (GPX4) and activate long-chain acyl-CoA synthase 4 (ACSL4), resulting in lipid ROS accumulation and subsequent exacerbation of ovarian reserve decline^26^. In a related study, Schröder et al. found that in estrogen receptor 1 (Esr1)-deficient mice, the expression of lipid transport protein 2 (Ltp2) was increased in the ovaries^27^. This led to iron metabolism disorders and foam cell formation, thereby exacerbating ovarian aging^27^.

**4.4 Disorder of Nucleotide Metabolism**

Abnormal nucleotide metabolism contributes to ovarian aging by affecting energy supply and redox balance. Yang et al. found that levels of NAD+ significantly decrease with age, thereby inhibiting SIRT2/3 activity and disrupting mitochondrial energy metabolism homeostasis^30^. NAD+ deficiency has also been demonstrated to inhibit PARP1-mediated DNA repair, thereby creating a self-perpetuating cycle^31^. Furthermore, upregulation of CD38 expression has been demonstrated to accelerate NAD+ depletion, thereby further exacerbating mitochondrial dysfunction and oocyte quality decline^30^. The findings of these studies suggest a direct correlation between nucleotide metabolism disorders and ovarian aging, with implications for energy metabolism and DNA repair.

**4.5 Disorder of Amino Acid Metabolism**

The association between amino acid metabolism abnormalities and ovarian aging is a subject that is being increasingly elucidated. Camaioni et al. observed that a disturbance in protein homeostasis during the process of ovarian aging, particularly the functional decline of longevity proteins, directly impacts oocyte quality and mitochondrial function^32^. Harasimov et al. discovered that an atypical buildup of proteins associated with longevity in the ovaries results in elevated levels of endoplasmic reticulum stress and subsequent granulosa cell apoptosis^33^. Furthermore, PRDX4 deficiency has been shown to disrupt disulfide bond formation, reduce FSHR expression, and further disrupt ovarian hormone synthesis^34^.

**Section 5 Extracellular matrix remodeling and changes in the immune microenvironment**

**5.1 Dynamic regulation of macrophages**

Studies have shown that macrophages (MFs) play an important role in maintaining ovarian homeostasis during folliculogenesis, follicular ovulation, luteolysis and decline, as well as in ovarian tissue homeostasis and immune surveillance^35,36^. Chuanchuan Z et al. found that activation of the apoptotic pathway in MFs increased with age^37^, resulting in a shift from an immunomodulatory microenvironment involving tissue-resident macrophages in young ovaries to a pro-inflammatory microenvironment involving macrophages of focal monocyte origin in middle-aged ovaries^37^. Shen H H et al. also showed that along with increased levels of inflammatory factors and apoptotic cellular macrophages, phagocytic activity is progressively impaired^36^. This altered ovarian immune microenvironment further promotes stromal cell senescence and accelerates reproductive decline^37^.

These studies have shown that as women age, the ovarian tissue microenvironment changes and manifests itself in chronic inflammation and increased ovarian fibrosis. Macrophages exhibit highly dynamic responses to specific danger signals in aging ovaries^37^. As illustrated in Supplementary Figure 3, macrophages respond to specific danger signals within the aging ovary by polarising ovarian MFs into different M1 or M2 phenotypes in response to tissue-derived stimuli such as inflammatory cytokines and Th2-type cytokines, and play a unique function in ovarian aging^35^. It was found that the M1 subpopulation plays an important role in maintaining follicular development and ovarian physiological elements and is mainly involved in primordial follicle activation, whereas the M2 phenotype is mainly involved in maintaining follicular dormancy^35^. The M1/M2 phenotype is not invariable at different stages of ovarian aging. In the early stages, the M1 phenotype predominates and acts as a pro-inflammatory agent^35^. In contrast, in ovarian aging, DAMPs (e.g., mtDNA) activate the M1-type NF-κB pathway via TLR4, whereas IL-4 induces M2-type polarization via STAT6^37^. M2 macrophages secrete TGF-β1, which promotes collagen deposition via SMAD3 phosphorylation, leading to ovarian fibrosis^35^. Thus, ovarian senescence is likely to be associated with an increased M2 phenotype in the later stages and is bound to affect the progression of ovarian senescence if the process of macrophages responding to specific danger signals within the aging ovary goes awry.

**5.2 Synergistic effects of other immune cells**

In addition to macrophages, other immune cells, such as neutrophils and T cells, have been shown to play a pivotal role in the aging process of the ovary. A body of research suggests a link between neutrophil activity and the exacerbation of oxidative stress and inflammatory responses in the ovaries^26^. Chen et al. found that this exacerbation is believed to be caused by the release of neutrophil extracellular traps (NETs) and reactive oxygen species (ROS) by these cells. In aged ovaries, there is an increase in neutrophil infiltration, which promotes the release of inflammatory factors such as IL-1β and TNF-α^26,38^. These factors further impair oocyte quality and the follicular microenvironment. Furthermore, excessive neutrophil activation has been demonstrated to result in ovarian tissue damage and accelerate the fibrotic process^26,39^.

Imbalance in T cell subsets is also a hallmark of ovarian aging. CD4+ T cells polarize toward the pro-inflammatory Th1 phenotype, promoting ovarian fibrosis and granulosa cell apoptosis by secreting IFN-γ^40^. Conversely, regulatory T cells (Tregs) undergo a decline in both number and function during the aging process^40^. This decline results in impaired immune suppression and increased susceptibility to autoimmune attacks on the ovaries^40^. Furthermore, aberrant activation of CD8+ cytotoxic T cells has been observed to directly induce oocyte destruction via the granzyme B (Granzyme B) pathway, thereby expediting the depletion of ovarian reserve^40^.

The role of natural killer (NK) cells in ovarian aging is also gradually being revealed. Research has demonstrated that the cytotoxic activity of natural killer (NK) cells in aged ovaries is augmented, potentially inducing granulocyte apoptosis via the Fas/FasL pathway, thereby affecting follicle development^41^. Concurrently, the abnormal activation of B cells may lead to the production of autoantibodies (e.g., anti-ovarian antibodies), which can further exacerbate ovarian functional decline^41^. The synergistic actions of these immune cells collectively shape the pro-inflammatory microenvironment of aged ovaries, accelerating the decline in reproductive function.

**Section 6 Therapeutic target for ovarian aging**

**6.1 Stem cell therapy**

Mesenchymal stem cells (MSCs) have multidirectional differentiation and can be isolated from a wide range of tissues such as umbilical cord, placenta, bone marrow, etc^42,43^. Many studies have shown that estrous cycle, ovarian weight, primordial follicle number and proportion, granulosa cell proliferation and angiogenesis can be improved after orthotopic transplantation of MSCs into the ovary. In addition, Wendi Pei et al. showed that the tumourigenesis, acute toxicity, immunogenicity and biodistribution of MSCs^44,45^, and the results showed that MSC therapy has a high degree of safety and efficacy.

A plethora of stem cells have been identified as potential candidates for the purpose of decelerating ovarian aging. Nan L et al. discovered that the transplantation of Human Umbilical Cord Mesenchymal Stem Cells-Derived Extracellular Vesicles (hucMSC-EVs) was capable of reversing premature ovarian failure in mice^46^. The investigation revealed that the process of premature ovarian failure led to a decrease in the levels of p-PI3K/PI3K and p-Akt/Akt, resulted in the inhibition of GC growth and even the induction of apoptosis, which culminated in significant oocyte loss^46^. Treatment with hucMSC-EVs has been demonstrated to elicit a number of significant biological responses, including the activation of the PI3K/Akt pathway, the promotion of the proliferation of ovarian GCs, and the inhibition of their apoptosis^46^. The therapeutic effect of hucMSC-EVs has also been verified in human ovarian granulosa cell lines in vitro^46^. Congcong G et al. found that transplantation of bone marrow mesenchymal stem cells (BMSCs) could help restore fertility in an animal model of POF^42^. Furthermore, Lu G et al. further demonstrated that the combination of BMSCs and moxibustion exhibited superior efficacy^47^. Moxibustion increased the expression of Sdf1 and Cxcr4, promoted the migration of BMSCs, enhanced the migration and homing of BMSCs after transplantation, and improved their ability to repair ovarian damage^47^. The combination of BMSCs and moxibustion reduced ROS levels, reduced mitotic overactivation, prevented mitochondrial damage and ultimately improved ovarian function^47^. Huang B et al. found that transplantation of human amniotic fluid mesenchymal stem cells (hAFMSCs) increased the mRNA and protein expression levels of ovarian markers in the four phases of folliculogenesis^48^. The study also found that the transplantation inhibited the expression of DNA-damage genes and improved the function of mouse ovaries^48^. Furthermore, studies have identified the therapeutic potential of adipose-derived MSCs, human menstrual blood-derived MSCs, ovarian hormone stem cells, and chorionic template MSC transplants^42,49,50^.

**6.2 Hormone Replacement Therapy (HRT)**

The fundamental mechanism of HRT for ovarian aging is to enhance the ovarian microenvironment and decelerate follicular depletion through exogenous hormone supplementation. Microvilli are critical for oocyte-granulosa cell communication, and E2 preserves oocyte health by maintaining the number of microvilli^51^. Concurrently, E2 inhibits the secretion of GnRH pulse in the hypothalamus through a negative feedback loop, leading to a reduction in elevated FSH/LH levels and, consequently, a decrease in the overstimulation of the residual follicles^52^. In addition, E2 increased local VEGF expression in the ovary, thereby improving ovarian blood supply and delaying interstitial fibrosis^53^. Conversely, cyclically added progesterone inhibited aberrant follicular activation by modulating the intra-ovarian progesterone receptor (PR) and reduced the release of pro-inflammatory factors to further protect the ovarian tissue^54^.

**6.3 Antioxidant therapy**

Researches have demonstrated that the use of antioxidants as a safe and effective adjunctive therapy for women experiencing ovarian aging is well-supported by the extant literature^55^. There are also many types of antioxidants that can be used to treat ovarian aging.

Resveratrol is an anti-aging, antioxidant and anti-inflammatory natural polyphenol compound^56^. Xu et al. found that resveratrol regulates mitochondrial biogenesis and degradation through the SIRT1 pathway, thereby controlling oocyte mitochondrial mass and attenuating the inhibition of oocyte maturation by nicotinamide, a specific inhibitor of sirtuins^57^. Hongyan Z et al. found that with age, the expression of SIRT1 and NRF2 decreases, and the levels of pro-inflammatory cytokines and endoplasmic reticulum stress markers GRP78 and CHOP increase, whereas resveratrol activates SIRT1/NRF2 to reduce inflammation and endoplasmic reticulum stress, thereby in a slowing down ovarian aging in a short period of time^58^.

HSYC is a traditional Chinese herbal formula and has been clinically proven to have an anti-aging effect on the ovaries^59^. Liuqing Y et al. found that HSYC supplementation eliminated the accumulation of ROS over time and inhibited DNA damage and autophagy during oocyte maturation in vitro^59^. Meanwhile, the expression level of SIRT3 was up-regulated after HSYC treatment, mitochondrial membrane potential was increased, Ca^2+^ level was decreased, and mitochondrial function could be improved^59^. Moreover, HSYC treatment resulted in a sustained increase in the expression levels of superoxide dismutase (SOD), PCG1α, and TFAM, whereas the acetylation level of SOD was reduced, enhancing the antioxidant function^59^.

Yang et al. found that echinacoside (ECH) could enhance mitochondrial function and reduce the excessive accumulation of ROS by activating SIRT1^60^. Concurrently, ECH increased the activities of antioxidant enzymes, such as SOD and catalase (CAT)^60^. These enzymes could effectively neutralize the free radicals and alleviate the damage caused by oxidative stress to oocytes^60^. Furthermore, ECH restored the mitochondrial membrane potential, enhanced the efficiency of ATP generation, and further reduced the generation of ROS^60^. In regard to oocyte quality, ECH demonstrated a substantial reduction in spindle abnormalities and chromosome misalignment during meiosis^60^. Additionally, it enhanced the proportion of mature oocytes and diminished the activity of senescence-related markers^60^. Consequently, ECH has the potential to enhance its antioxidant effects through multiple pathways, suggesting a promising avenue for the treatment of ovarian aging.

**6.4 Growth hormone (GH)**

GH is mainly secreted by eosinophils in the anterior pituitary gland and promotes sexual maturation and gonadal cell proliferation, improves ovarian response to gonadotropins, promotes follicular development and maturation, regulates steroid secretion, improves endometrial receptivity and improves embryo quality^61^. Lei H et al. found that GH has a well-documented function in delaying ovarian aging, and the mechanism involves the role of GH in maintaining meiosis in the oocyte and influencing the maturation of the oocyte nucleus^61^. It has been shown that GH directly affects ovarian function by binding to GHR on the ovary, and the interaction between GH and GHR is mediated by cAMP and PKA, which promotes follicular growth, maturation and ovulation, inhibits follicular atresia and controls late-stage follicle formation^61^. In addition, GH indirectly regulates ovarian function through the IGF system, which plays a role in promoting early folliculogenesis, increasing follicular sensitivity to gonadotropins, accelerating oocyte nuclear maturation, and improving mitochondrial activity and oocyte quality^61^.

It is evident that although GH does not completely inhibit apoptosis in early antral follicles, it enhances proliferation, inhibits apoptosis, and promotes expansion of cumulus cells to maintain meiosis in oocytes and affects maturation of the oocyte nucleus^61^.

**6.5 Maintenance of homeostasis**

Mitochondrial dysfunction is one of the important factors in ovarian aging. Maintaining mitochondrial functional homeostasis improves energy metabolism in oocytes and reduces oxidative stress damage, thereby delaying ovarian aging. It was found that the traditional Chinese medicine EZTG could regulate mitochondrial homeostasis, inhibit iron death, and delay ovarian aging by maintaining PINK1/parkin-mediated mitochondrial homeostasis, decreasing lipid peroxidation caused by ROS accumulation, and inhibiting iron pituitary diseases^62^. Zhicheng J et al. found experimentally that EZTG gavage treatment improved the ovaries of aged mice, resulting in an increase in reserve; improved serum hormone levels; increased levels of mitochondrial membrane potential and the lipid peroxidation marker glutathione in the ovaries; decreased levels of ROS and mitochondrial DNA damage markers; decreased levels of PINK1 and parkin; and improved mitochondrial morphology; Decreased total iron and protein levels in ACSL4; increased protein levels in GPX4^62^.

Several studies have demonstrated that the ovary has more very long-lived proteins compared to other tissues^33^. Harasimov K et al. showed that these long-lived proteins have multiple functions, including mitochondrial, cytoskeletal, chromatin, and proteostasis, and protein homeostasis in the ovary is tied to the longevity of these proteins^33^. It is evident that PRDX4 plays a pivotal regulatory role in maintaining protein homeostasis, with a particular emphasis on long-lived proteins^34^. A study by Xiaofei Zou et al. found that a lack of PRDX4 leads to dysregulation of protein homeostasis, increased ER stress, and increased granulosa cell apoptosis, ultimately leading to accelerated ovarian decline^34^. In addition, it was found that due to impaired disulfide bond formation, the expression of FSHR, was significantly decreased in old mice, especially functional trimers^34^. In contrast, overexpression of PRDX4 promotes the maintenance of protein homeostasis and reduces ER stress, thereby increasing E2 levels^34^. Thus, the relevant regulation of PRDX4 expression becomes a novel target for the treatment of ovarian aging.

**Section 7 Discussion**

The conclusions of several of the studies included in this review are contradictory, but there are some plausible explanations for this phenomenon. For instance, certain studies have indicated that ROS may directly induce oocyte damage, while others have suggested that ROS may function as cell signaling molecules. The present study hypothesizes that the role of ROS is concentration-dependent. The physiological levels of ROS are involved in signaling; however, excessive accumulation can lead to oxidative stress. However, the diminished antioxidant capacity observed in aged ovaries renders them more susceptible to ROS-induced damage to oocytes. Furthermore, with regard to the therapeutic efficacy of antioxidant treatment protocols for ovarian aging, conflicting conclusions have emerged from research studies. While some studies have indicated that antioxidants exhibit a more pronounced therapeutic effect, others have suggested that the utilization of antioxidants disrupts physiological ROS (reactive oxygen species) signaling. The efficacy of antioxidants is contingent upon the temporal and quantitative parameters of the intervention. Oxidative stress is already imbalanced in aging ovaries, and it is more important to precisely regulate rather than merely inhibit ROS.

Despite the comprehensive nature of the review, it is important to note that many of the study's key findings are still in the preliminary stages. The most salient issue in the mechanisms section is that the majority of mechanisms have been studied based on mouse models only. However, human ovarian aging may not be identical. To further substantiate the findings derived from animal models, it is imperative to enhance the research on mechanisms such as BRCA1 mutation, telomere shortening, m6A modification, and iron death through multi-omics analysis of human ovarian tissue samples. Furthermore, extant studies on mechanisms such as NAD+/SIRT2 or mitochondrial oxidative stress have predominantly focused on a single pathway. However, ovarian aging is the result of synergistic effects of multiple mechanisms. For instance, further elucidation is necessary to fully comprehend the impact of the inflammatory microenvironment on mitochondrial function and the role of epigenetic modifications before a more systematic molecular network model can be developed. Secondly, there remains an absence of dynamic tracking data for specific stage-specific mechanisms. For instance, we hypothesized that DNA damage dominates in the early stage, while metabolic disorders dominate in the late stage of the disease. Longitudinal studies are necessary to elucidate the hallmark events and their causal relationships in different stages.

**Reference**

1. Santoro N, Roeca C, Peters BA, Neal-Perry G. The Menopause Transition: Signs, Symptoms, and Management Options. *The Journal of Clinical Endocrinology & Metabolism*. 2021;106(1):1-15.

2. Shi L, Wang H, Zhu S, et al. Multi-Omics Reveal the Metabolic Changes in Cumulus Cells During Aging. *Cell Proliferation*. 2025;n/a(n/a):e70014.

3. Albamonte MI, Albamonte MS, Bou-Khair RM, Zuccardi L, Vitullo AD. The ovarian germinal reserve and apoptosis-related proteins in the infant and adolescent human ovary. *J Ovarian Res*. 2019;12(1):22.

4. Hoyt LT, Falconi AM. Puberty and perimenopause: reproductive transitions and their implications for women’s health. *Soc Sci Med*. 2015;132:103-112.

5. Paramsothy P, Harlow SD, Nan B, et al. Duration of the menopausal transition is longer in women with young age at onset: the multiethnic Study of Women’s Health Across the Nation. *Menopause*. 2017;24(2):142-149.

6. Lawley SD, Johnson J. Why is there an “oversupply” of human ovarian follicles?†. *Biol Reprod*. 2023;108(5):814-821.

7. Depmann M, Faddy MJ, van der Schouw YT, et al. The Relationship Between Variation in Size of the Primordial Follicle Pool and Age at Natural Menopause. *J Clin Endocrinol Metab*. 2015;100(6):E845-851.

8. Lawley SD, Sammel MD, Santoro N, Johnson J. Mathematical recapitulation of the end stages of human ovarian aging. *Sci Adv*. 2024;10(2):eadj4490.

9. Jiang Z xin, Wang Y ning, Li Z yuan, et al. The m6A mRNA demethylase FTO in granulosa cells retards FOS-dependent ovarian aging. *Cell Death Dis*. 2021;12(8):1-10.

10. Li P, Lin Y, Ma H, et al. Epigenetic regulation in female reproduction: the impact of m6A on maternal-fetal health. *Cell Death Discov*. 2025;11(1):1-30.

11. Mani S, Srivastava V, Shandilya C, Kaushik A, Singh KK. Mitochondria: the epigenetic regulators of ovarian aging and longevity. *Front Endocrinol*. 2024;15.

12. Qian C, Liu Z, Qian Y, et al. Increased N6-methyladenosine is related to the promotion of the methyltransferase METTL14 in ovarian aging. *Genes Dis*. 2024;11(3):101050.

13. Li L, Yang L, Shen L, Zhao Y, Wang L, Zhang H. Fat Mass and Obesity-Associated Protein Regulates Granulosa Cell Aging by Targeting Matrix Metalloproteinase-2 Gene Via an N6-Methyladenosine-YT521-B Homology Domain Family Member 2-Dependent Pathway in Aged Mice. *Reprod Sci*. 2024;31(11):3498-3511.

14. Kong L, Gong Y, Wang Y, et al. Multi-omics revealed that DCP1A and SPDL1 determine embryogenesis defects in postovulatory ageing oocytes. *Cell Proliferation*. 2025;58(3):e13766.

15. Clark H, Knapik LO, Zhang Z, et al. Dysfunctional MDR-1 disrupts mitochondrial homeostasis in the oocyte and ovary. *Sci Rep*. 2019;9(1):9616.

16. Chiang JL, Shukla P, Pagidas K, et al. Mitochondria in Ovarian Aging and Reproductive Longevity. *Ageing Res Rev*. 2020;63:101168.

17. Nabi D, Bosi D, Gupta N, Thaker N, Fissore R, Brayboy LM. Multidrug resistance transporter-1 dysfunction perturbs meiosis and Ca2+ homeostasis in oocytes. *Reproduction*. 2023;165(1):79-91.

18. Taneja C, Gera S, Kim SM, Iqbal J, Yuen T, Zaidi M. FSH-metabolic circuitry and menopause. *J Mol Endocrinol*. 2019;63(3):R73-R80.

19. Chu YL, Xu YR, Yang WX, Sun Y. The role of FSH and TGF-β superfamily in follicle atresia. *Aging (Albany NY)*. 2018;10(3):305-321.

20. Bhartiya D, Singh J. FSH-FSHR3-stem cells in ovary surface epithelium: basis for adult ovarian biology, failure, aging, and cancer. *Reproduction*. 2015;149(1):R35-48.

21. Zaidi M, Lizneva D, Kim SM, et al. FSH, Bone Mass, Body Fat, and Biological Aging. *Endocrinology*. 2018;159(10):3503-3514.

22. Broekmans FJ, Soules MR, Fauser BC. Ovarian aging: mechanisms and clinical consequences. *Endocr Rev*. 2009;30(5):465-493.

23. Tiwari-Pandey R, Ram Sairam M. Modulation of ovarian structure and abdominal obesity in curcumin- and flutamide-treated aging FSH-R haploinsufficient mice. *Reprod Sci*. 2009;16(6):539-550.

24. Zhang Y, Zhang D. FSH/FSHR/TGF-β1/SMADS SIGNALING CASCADE MEDIATES OVARIAN FIBROSIS RESULTING IN OVARIAN AGING. *Fertility and Sterility*. 2023;120(4, Supplement):e33.

25. Ma J, Chen S, Liu J, et al. Cryptochrome 1 regulates ovarian granulosa cell senescence through NCOA4-mediated ferritinophagy. *Free Radic Biol Med*. 2024;217:1-14.

26. Chen Y, Zhang J, Tian Y, et al. Iron accumulation in ovarian microenvironment damages the local redox balance and oocyte quality in aging mice. *Redox Biol*. 2024;73:103195.

27. Schröder SK, Krizanac M, Kim P, Kessel JC, Weiskirchen R. Ovaries of estrogen receptor 1-deficient mice show iron overload and signs of aging. *Front Endocrinol (Lausanne)*. 2024;15:1325386.

28. ZHU N. Effect of advanced glycosylation end products (AGEs) on the quiescence maintenance or activation of primordial follicle. In: *Abstracts Collection of Papers from the 5th World Congress of Integrative Medicine 2017 (Next Book)*. 2017:1.

29. Wu J, Zhao X, Fang Y, et al. GPD1L-Mediated Glycerophospholipid Metabolism Dysfunction in Women With Diminished Ovarian Reserve: Insights From Pseudotargeted Metabolomic Analysis of Follicular Fluid. *Cell Proliferation*. 2025;n/a(n/a):e70024.

30. Yang Q, Chen W, Cong L, et al. NADase CD38 is a key determinant of ovarian aging. *Nat Aging*. 2024;4(1):110-128.

31. Liang J, Huang F, Song Z, Tang R, Zhang P, Chen R. Impact of NAD+ metabolism on ovarian aging. *Immun Ageing*. 2023;20(1):70.

32. Camaioni A, Ucci MA, Campagnolo L, De Felici M, Klinger FG, Italian Society of Embryology, Reproduction and Research (SIERR). The process of ovarian aging: it is not just about oocytes and granulosa cells. *J Assist Reprod Genet*. 2022;39(4):783-792.

33. Harasimov K, Gorry RL, Welp LM, et al. The maintenance of oocytes in the mammalian ovary involves extreme protein longevity. *Nat Cell Biol*. 2024;26(7):1124-1138.

34. Zou X, Liang X, Dai W, et al. Peroxiredoxin 4 deficiency induces accelerated ovarian aging through destroyed proteostasis in granulosa cells. *Biochim Biophys Acta Mol Basis Dis*. 2024;1870(7):167334.

35. Tang M, Zhao M, Shi Y. New insight into the role of macrophages in ovarian function and ovarian aging. *Front Endocrinol (Lausanne)*. 2023;14:1282658.

36. Shen HH, Zhang XY, Liu N, et al. Chitosan alleviates ovarian aging by enhancing macrophage phagocyte-mediated tissue homeostasis. *Immun Ageing*. 2024;21(1):10.

37. Zhou C, Guo Q, Lin J, et al. Single-Cell Atlas of Human Ovaries Reveals The Role Of The Pyroptotic Macrophage in Ovarian Aging. *Adv Sci (Weinh)*. 2024;11(4):e2305175.

38. Amargant F, Manuel SL, Tu Q, et al. Ovarian stiffness increases with age in the mammalian ovary and depends on collagen and hyaluronan matrices. *Aging Cell*. 2020;19(11):e13259.

39. Wu M, Tang W, Chen Y, et al. Spatiotemporal transcriptomic changes of human ovarian aging and the regulatory role of FOXP1. *Nat Aging*. 2024;4(4):527-545.

40. Nikolich-Žugich J. The twilight of immunity: emerging concepts in aging of the immune system. *Nat Immunol*. 2018;19(1):10-19.

41. Solana R, Tarazona R, Gayoso I, Lesur O, Dupuis G, Fulop T. Innate immunosenescence: effect of aging on cells and receptors of the innate immune system in humans. *Semin Immunol*. 2012;24(5):331-341.

42. Guo C, Ma Y, Situ Y, et al. Mesenchymal stem cells therapy improves ovarian function in premature ovarian failure: a systematic review and meta-analysis based on preclinical studies. *Front Endocrinol (Lausanne)*. 2023;14:1165574.

43. Lee DR, Lee JE. Preservation of ovarian function using human pluripotent stem cell-derived mesenchymal progenitor cells. *Clin Exp Reprod Med*. Published online May 24, 2024.

44. Pei W, Fu L, Guo W, et al. Efficacy and safety of mesenchymal stem cell therapy for ovarian ageing in a mouse model. *Stem Cell Res Ther*. 2024;15(1):96.

45. Yan L, Tu W, Zhao X, et al. Stem cell transplantation extends the reproductive life span of naturally aging cynomolgus monkeys. *Cell Discov*. 2024;10(1):1-17.

46. Li N, Fan X, Liu L, Liu Y. Therapeutic effects of human umbilical cord mesenchymal stem cell-derived extracellular vesicles on ovarian functions through the PI3K/Akt cascade in mice with premature ovarian failure. *Eur J Histochem*. 2023;67(3):3506.

47. Lu G, Li HX, Song ZW, et al. Combination of bone marrow mesenchymal stem cells and moxibustion restores cyclophosphamide-induced premature ovarian insufficiency by improving mitochondrial function and regulating mitophagy. *Stem Cell Res Ther*. 2024;15(1):102.

48. Huang B, Ding C, Zou Q, Lu J, Wang W, Li H. Human Amniotic Fluid Mesenchymal Stem Cells Improve Ovarian Function During Physiological Aging by Resisting DNA Damage. *Front Pharmacol*. 2020;11:272.

49. Shen L, Liu J, Luo A, Wang S. The stromal microenvironment and ovarian aging: mechanisms and therapeutic opportunities. *J Ovarian Res*. 2023;16(1):237.

50. Hirakawa T, Yotsumoto F, Imi S, et al. Adipose-derived Mesenchymal Stem Cell Transplantation Improves Ovarian Function and Oocyte Quality in Aged Mice. *Anticancer Res*. 2024;44(8):3577-3586.

51. Yang P, Wang J, Shen Y, Roy SK. Developmental Expression of Estrogen Receptor (ER) α and ERβ in the Hamster Ovary: Regulation by Follicle-Stimulating Hormone.

52. Nelson LM. Clinical practice. Primary ovarian insufficiency. *N Engl J Med*. 2009;360(6):606-614.

53. Kalantaridou SN, Naka KK, Papanikolaou E, et al. Impaired endothelial function in young women with premature ovarian failure: normalization with hormone therapy. *J Clin Endocrinol Metab*. 2004;89(8):3907-3913.

54. ESHRE, ASRM, CREWHIRL and IMS Guideline Group on POI, Panay N, Anderson RA, et al. Evidence-based guideline: Premature Ovarian Insufficiency. *Fertil Steril*. 2025;123(2):221-236.

55. Shang Y, Song N, He R, Wu M. Antioxidants and Fertility in Women with Ovarian Aging: A Systematic Review and Meta-Analysis. *Adv Nutr*. 2024;15(8):100273.

56. Gou M, Li J, Yi L, et al. Reprogramming of ovarian aging epigenome by resveratrol. *PNAS Nexus*. 2023;2(2):pgac310.

57. Xu D, ZHAO Z, ZHAO Y. Mechanism of NAD+/SIRT2 Pathway Regulating Mature Quality of Aged Oocytes. *Acta Veterinaria et Zootechnica Sinica*. 2022;53(6):1657-1667.

58. Zhu H, Li X, Qiao M, Sun X, Li G. Resveratrol Alleviates Inflammation and ER Stress Through SIRT1/NRF2 to Delay Ovarian Aging in a Short-Lived Fish. *J Gerontol A Biol Sci Med Sci*. 2023;78(4):596-602.

59. Yang L, Shang J, Wang H, et al. Promising anti-ovarian aging herbal formulation He’s Yangchao promotes in vitro maturation of oocytes from advanced maternal age mice. *J Ethnopharmacol*. 2024;318(Pt A):116890.

60. Yang L, Lai X, Lin F, et al. Revitalising Aging Oocytes: Echinacoside Restores Mitochondrial Function and Cellular Homeostasis Through Targeting GJA1/SIRT1 Pathway. *Cell Proliferation*. 2025;n/a(n/a):e70044.

61. Han L, Tian H, Guo X, Zhang L. Regulation of ovarian function by growth hormone: Potential intervention of ovarian aging. *Front Endocrinol (Lausanne)*. 2022;13:1072313.

62. Zhicheng J, Yongqian L, Peixuan W, et al. ErZhiTianGui Decoction alleviates age-related ovarian aging by regulating mitochondrial homeostasis and inhibiting ferroptosis. *J Ovarian Res*. 2024;17(1):12.

**FIGURES**

Supplementary Figure 1 | Mechanisms of mitochondrial disorders leading to ovarian senescence. (By Figdraw.)

The lack of protective histones in mitochondrial mtDNA and electron leakage from the mitochondrial respiratory chain makes mitochondria more susceptible to excessive ROS-induced oxidative stress damage. The accumulation of ROS leads to a decrease in the copy number and function of mitochondrial DNA in the ovary, resulting in follicular atresia. It also damages the ovarian microenvironment, accelerating follicular atrophy, reducing oocyte quality and ultimately leading to ovarian failure.

Supplementary Figure 2 | Iron metabolism contributes to ovarian aging. (By Figdraw.)

Increased iron content and abnormal expression of iron-metabolising proteins in senescent ovaries and oocytes, senescent oocytes showing greater ferritin phagocytosis and sustained increases in cell membrane Fe^2+^, increased lipid peroxidation, mitochondrial dysfunction and increased lysosomal activity lead to disruption of senescence-associated iron homeostasis in the ovary, resulting in excessive ROS production and reduced ovarian reserve and oocyte quality in aging mice.

Supplementary Figure 3 | The process of macrophage polarisation in response to specific danger signals within the aging ovary. (By Figdraw.)

In response to tissue-derived stimuli such as cytokines, ovarian MFs undergo a process of polarisation, resulting in the formation of distinct M1 or M2 phenotypes. Furthermore, the M1/M2 phenotype exhibits distinct characteristics at varying stages of ovarian aging. During the initial stages, the M1 phenotype is predominantly pro-inflammatory and involved in the activation of primordial follicles. Conversely, in the later stages, the M2 phenotype is more prevalent and plays a role in maintaining follicular dormancy, which can potentially lead to ovarian fibrosis.
