# Supplementary figures and images for: Ovarian Aging: A Multifaceted Perspective on Mechanisms
Source: Cell Prolif. 2025 Nov 4;59(1):e70144. doi: 10.1111/cpr.70144 (PMC12774617; doi:10.1111/cpr.70144)

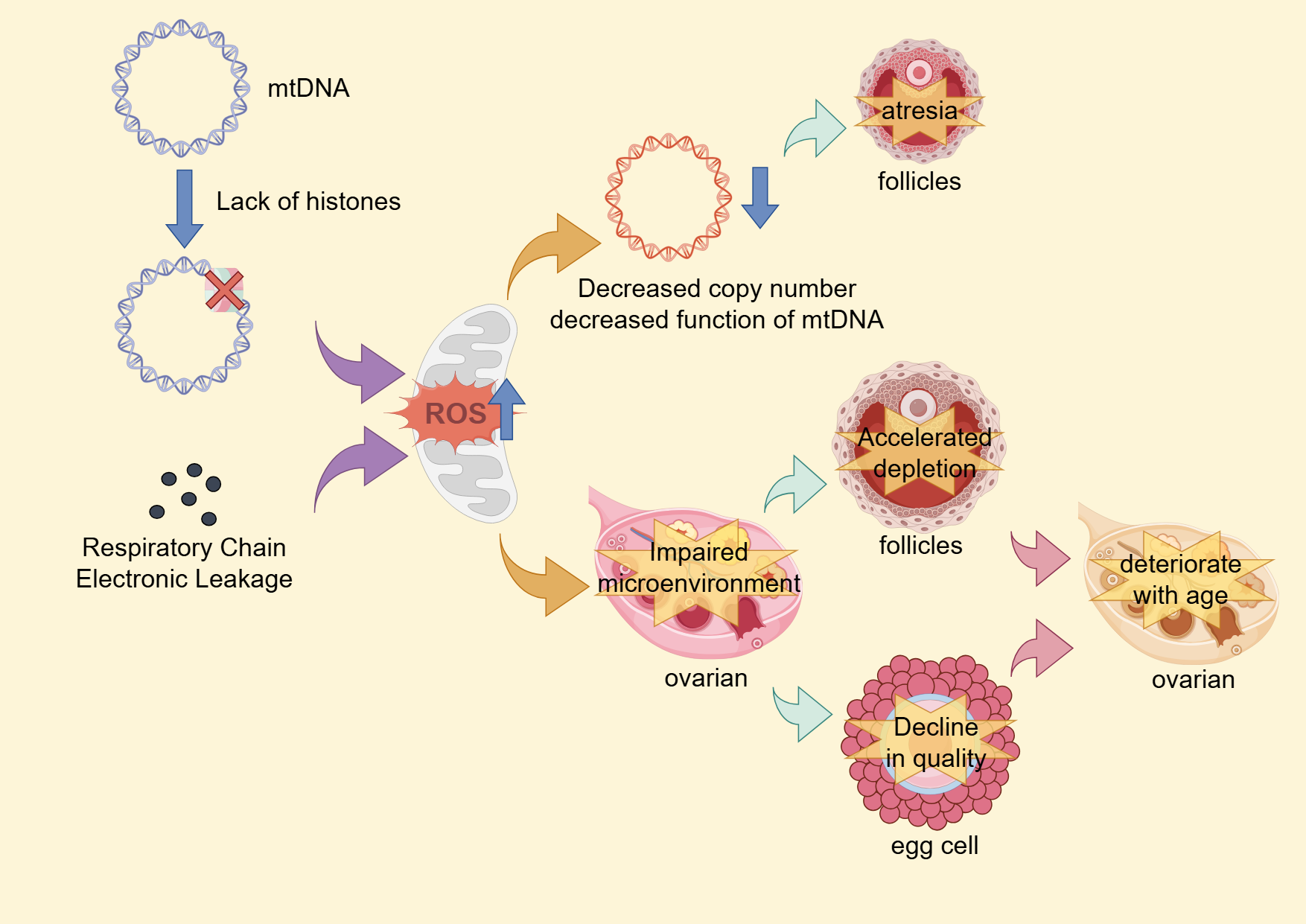

Supplement: Supplementary file 2 — Figure S1: Mechanisms of mitochondrial disorders leading to ovarian senescence. (By Figdraw). [file CPR-59-e70144-s002.png]

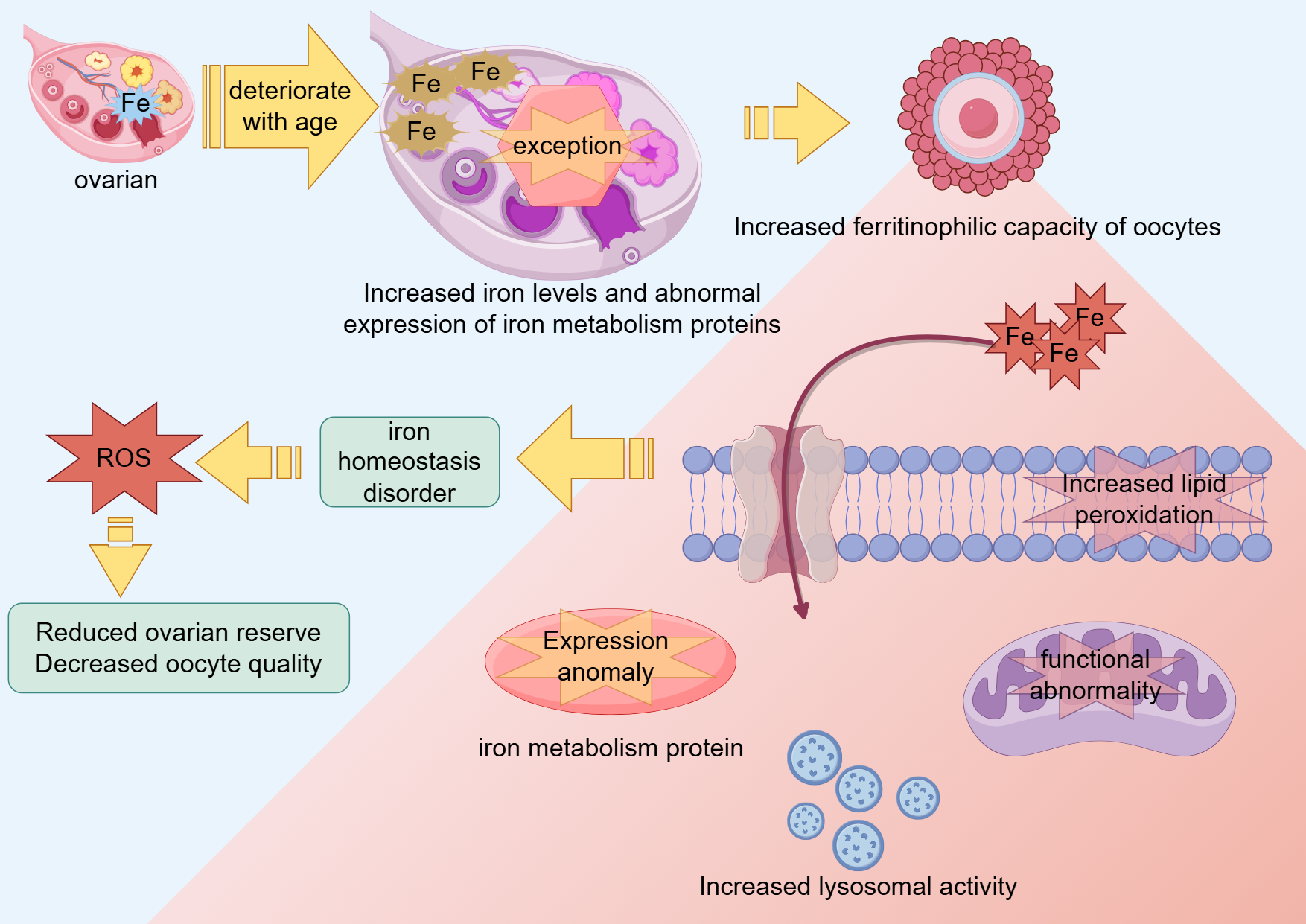

Supplement: Supplementary file 3 — Figure S2: Iron metabolism contributes to ovarian aging. (By Figdraw). [file CPR-59-e70144-s004.png]

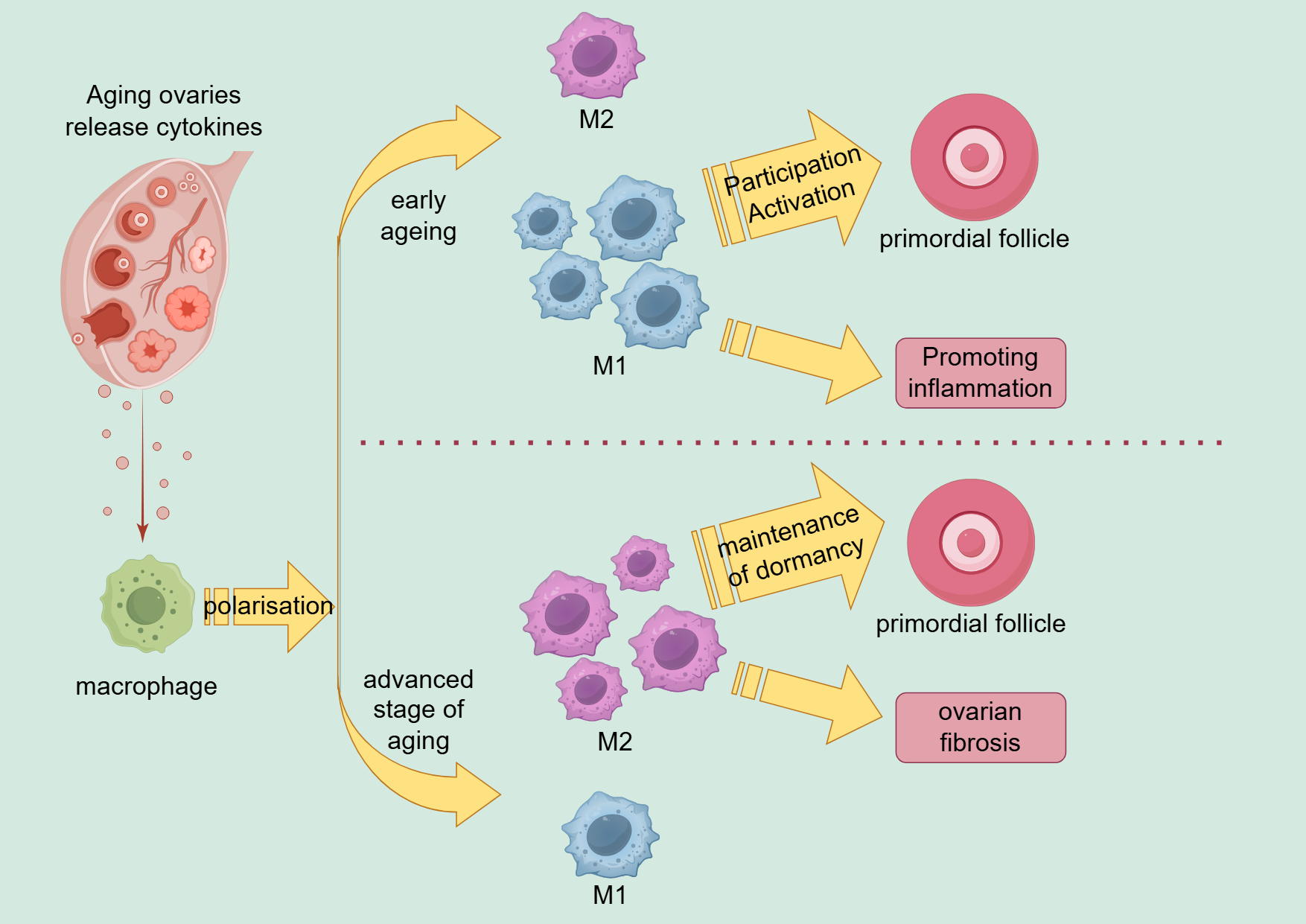

Supplement: Supplementary file 4 — Figure S3: The process of macrophage polarisation in response to specific danger signals within the aging ovary. (By Figdraw). [file CPR-59-e70144-s003.png]
